# Knockout of *Rlim* Results in a Sex Ratio Shift toward Males but Superovulation Cannot Compensate for the Reduced Litter Size

**DOI:** 10.3390/ani13061079

**Published:** 2023-03-17

**Authors:** Jingfeng Peng, Yunfei Hou, Shici Wu, Zicong Li, Zhenfang Wu

**Affiliations:** 1 National Engineering Research Center for Breeding Swine Industry, South China Agricultural University, Guangzhou 510642, China; 2 Department of Animal Genetics, Breeding and Reproduction, College of Animal Science, South China Agricultural University, Guangzhou 510642, China; 3 Guangdong Provincial Key Laboratory of Agro-Animal Genomics and Molecular Breeding, South China Agricultural University, Guangzhou 510642, China; 4 Guangdong Provincial Laboratory of Lingnan Modern Agricultural Science and Technology, Guangzhou 510642, China

**Keywords:** sex control, sex ratio, *Rlim*, X-chromosome inactivation, superovulation, litter size

## Abstract

**Simple Summary:**

Maternal *Rlim* is required for imprinted X-chromosome inactivation (XCI), which is essential for normal development of female mouse embryos. In this study, we used a novel approach to inactivate the maternal *Rlim* allele in female embryos, resulting in a male sex-ratio shift in the offspring. However, the reduced litter size caused by the loss of female embryos could not be compensated for by superovulation in the mother mice. The present study develops a new approach to select offspring sex according to the difference in *Rlim* function between male and female mouse embryos. This gender control approach may help to improve the productivity in livestock and prevent the sex-associated hereditary diseases in humans.

**Abstract:**

Technologies that can preselect offspring gender hold great promise for improving farm animal productivity and preventing human sex-related hereditary diseases. The maternal *Rlim* allele is required for imprinted X-chromosome inactivation, which is essential for the normal development of female mouse embryos. In this study, we inactivated the maternal *Rlim* allele in embryos by crossing a male transgenic mouse line carrying an X-linked *CMV-Cre* transgene with a female line carrying a *loxP*-flanked *Rlim* gene. Knockout of the maternal *Rlim* gene in embryos resulted in a male-biased sex ratio skew in the offspring. However, it also reduced litter size, and this effect was not compensated for by superovulation in the mother mice. In addition, we showed that siRNA-mediated knockdown of *Rlim* in mouse embryos leads to the birth of male-only progenies. This study provides a new promising method for male-biased sex selection, which may help to improve the productivity in livestock and prevent sex-associated hereditary diseases in humans.

## 1. Introduction

In farm animals, several economically important traits are associated with gender. For example, male animals usually have a fast growth rate and high feed conversion efficiency, while only females can give birth to progeny and produce milk [1,2]. In humans, some hereditary diseases are sex-linked [3,4]. Therefore, the development of technologies that can preselect the gender of the offspring can improve the production efficiency in animal husbandry and prevent sex-linked genetic disorders in humans.

Flow-cytometric sperm sorting separates the X- and Y chromosome-bearing sperm according to their DNA content. Using this technology, the predetermination of progeny sex has been achieved with about 90% accuracy in some livestock species such as horses, sheep, cattle and swine [5,6,7,8,9]. However, the cost of sperm sorting-based gender preselection technology is positively associated with the number of sorted sperm. Currently, this technology is mainly used in farm animals such as cattle, which require only a low dose of sperm for routine artificial insemination [10,11]. In contrast, sperm sorting is not feasible for livestock such as pigs which need a large number of sperm for successful artificial insemination [12,13,14]. It is therefore necessary to develop alternative gender predetermination technologies for livestock species that are unsuitable for flow-cytometric sperm sexing.

Recently, the CRISPR/Cas9 system has been employed for gender control in mice. Briefly, the sex ratio was skewed toward females by inactivating three vital genes for the early development of male embryos, via crossing a maternal transgenic mouse line carrying an autosome-inserted *Cas9* gene, with a paternal transgenic line carrying three small guide RNAs (sgRNAs) on the Y chromosome [15]. Male- or female-only offspring were produced by mating the father mice carrying a Y or X chromosome-linked Cas9 transgene with the mothers carrying an autosome-integrated sgRNA transgene, which targets the *Topoisomerase 1* (*Top1*) gene necessary for early embryonic survival [16]. A female-biased sex ratio shift was also observed in the progenies of male transgenic mice expressing a CRISPR/Cas9 system that cleaves the repetitive sequences on the Y chromosome [17]. These male transgenic mice produce only X sperm because their Y chromosome-bearing spermatogenic cells were eliminated during spermatogenesis by the CRISPR/Cas9 system targeting the Y chromosome.

**Figure 1:**
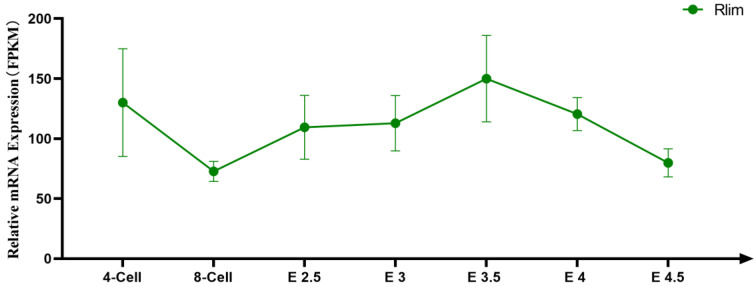
Schematic representation of *Rlim* mRNA expression level in female embryos during early developmental stages, from 4-cell stage to embryonic days 4.5. Data on Rlim mRNA expression levels in female embryos at early developmental stages were obtained from publicly available data from the study by Wang et al. [22].

*Rlim* is an X chromosome-linked gene that can trigger X chromosome inactivation (XCI) by activating the transcription of *Xist*, an XCI inducer [18,19]. *Rlim* is necessary for the normal development of female embryos, which express a relatively high level of *Rlim* during early embryonic stages (Figure 1). Deletion of the maternal *Rlim* allele in male mice and mutation of the paternal *Rlim* allele in female mice have no significant effect on their growth and fertility [20,21]. However, knockout of the maternal *Rlim* allele results in abnormal imprinted XCI in extraembryonic trophoblasts, which causes defective development of placentas and the death of female embryos in utero [18]. This regulatory mechanism suggests that mutation of the maternal *Rlim* allele can be used as a strategy to produce male-only offspring. It is important to note, however, that inactivating the maternal *Rlim* allele will reduce litter size due to the loss of female embryos.

Superovulation in the mothers may compensate for the reduction in litter size caused by mutating the maternal *Rlim* allele in female embryos, while still achieving a male-biased skew in the gender ratio. To test this hypothesis, in the present study we employed a new method to inactivate the maternal *Rlim* allele in mice, and investigated the combined effects of knockout of the maternal allele in the embryos and superovulation in the mother mice on offspring sex ratio and litter size.

## 2. Materials and Methods

### 2.1. gRNA Design and Synthesis, and Targeting Vector Construction

Two gRNAs (the sequences are shown in Table 1 and the targeting sites on the mouse *Rlim* gene are indicated in Figure 1) were designed according to the mouse *Rlim* gene sequences (NCBI Reference number: NM_001358205) and synthesized. A 1.52 kb 5′ homologous arm and a 1.53 kb 3′ homologous arm were generated by PCR amplification using a BAC clone (RP24-334J19) as a template. The two homologous arms were inserted into the SacI and the EcorV sites of a modified pUC57 plasmid (Cyagen, Guangzhou, China), respectively. The 2013 bp knockout region of the mouse *Rlim* gene, flanked by *loxP* sites, was synthesized and inserted between the SacI and EcorV sites of the above plasmid.

### 2.2. Microinjection of Fertilized Eggs

Fertilized eggs were harvested from the oviducts of 6- to 8-week-old C57BL/6J female mice that were mated with male mice from the same strain. The pronuclei of the harvested zygotes were microinjected with 1~2 pL of a solution containing 2 pmol/µL gRNA, 15 ng/µL targeting vector and 30 ng/µL Cas9 protein (Catalog #: M0646M; NEB, Beijing, China). The embryos were then transferred into the fallopian tubes of pseudo-pregnant female mice to obtain the F_0_ generation mice.

### 2.3. Genotype Identification by PCR

Genomic DNA was extracted from the tail biopsy samples of 3-week-old mice according to the manufacturer’s instructions (Omega, Norcross, GA, USA) and stored at −20 °C for later use. Four pairs of primers, including primer 1, primer 2, primers for pair 1 and primers for pair 2, were used to identify mice carrying the *Rlim*^fl/fl^ gene. The CMV-Cre primer pair was used to identify Cre-transgenic mice, while the SRY and ZFX primer pairs were used to identify the gender of mice that died during the perinatal period by double-primer PCR. The sequences of all primers are shown in Table 2.

### 2.4. Southern Blotting

The genomic DNA isolated from mouse tails was digested by Bsu 36I or Mfe I at 37 °C for 14–16 h according to the manufacturer’s instructions (NEB, Beijing, China). The digested DNA was purified by ethanol precipitation, separated by agarose gel electrophoresis, transferred to an Immobicon-Ny+ membrane (Catalog #: NG0312; GE Healthcare, Beijing, China) and fixed at 120 °C for 30 min. The 5′ Probe-Bsu36I and 3′ Probe-MfeI were synthesized according to the instructions of the PCR DIG probe synthesis Kit (Roche, Shanghai, China) and hybridized with the membrane. After hybridization, the membrane was incubated for 30 min in blocking solution and thereafter in anti-DIG-AP antibody solution (Roche, Shanghai, China) for 30 min. The membrane was subsequently exposed to 1 mL CSPD ready-to-use solution (Roche, Shanghai, China) for 5–20 min and photographs were taken using an imaging system.

### 2.5. CMV-Cre-Transgenic Male Mice

CMV-Cre-transgenic male mice with a C57BL/6J strain background were purchased from Guangzhou Cyagen Biotechnology Company (Cat. No. C001055, Guangzhou, China). The *Cre* transgene is integrated on the X chromosome and controlled by a CMV promoter.

### 2.6. Superovulation

Proestrus female mice aged 7–9 weeks were intraperitoneally injected with 10 IU PMSG (NSHF, Ningbo, China) and then with 10 IU HCG (NSHF, Ningbo, China) 48 h later.

### 2.7. Fetal Recovery

Superovulated 7- to 9-week-old female mice were mated with the Cre-transgenic male mice for one day, and the female mice with vaginal sperm plugs were moved to a separate cage. After 7.5–8.5 days of embryo development, the mice were euthanized by cervical dislocation and their uteri were removed by cesarean section to analyze fetal number and development.

### 2.8. Statistical Analysis

The distribution of the data was analyzed by a Kolmogorov–Smirnov test using SPSS 26.0. The difference in female to male ratio in the offspring transgenic mice was evaluated using a X^2^ test, and the difference in litter size and perinatal death rate among the different groups was evaluated using One-way ANOVA. GraphPad Prism 8.0 (GraphPad Software, San Diego, CA, USA) was used for all statistical analyses.

## 3. Results

### 3.1. Production and Identification of F_0_ Generation Rlim^fl^ Transgenic Male Mice and Rlim^fl/+^ Transgenic Female Mice

**Figure 2:**
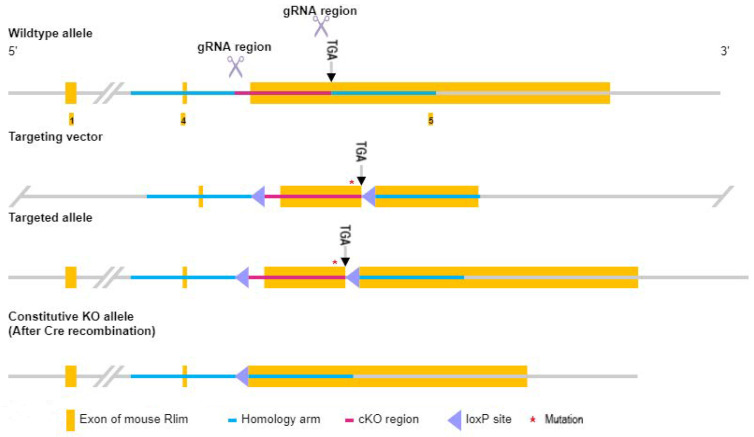
Schematic diagram of the strategy for knockout of the *Rlim* gene. Two sgRNAs were designed to direct the Cas9 enzyme to cut the wild-type *Rlim* allele and facilitate homologous recombination with the targeting vector. TGA is the stop codon site of the *Rlim* gene. A synonymous mutation was introduced into the targeting vector to avoid sgRNA-mediated cleavage of the targeting vector or the successfully targeted allele.

The strategy used in this study for knocking out the *Rlim* gene is shown in Figure 2. Briefly, a targeting vector was designed to flank a region in the mouse *Rlim* gene’s exon 5 with *loxP* sites. The targeting vector was used to generate a targeted *Rlim* allele using CRISPR/Cas9 in combination with homologous recombination. A CRE enzyme-induced recombination between the integrated two *loxP* sites results in the deletion of the floxed region of exon 5 of the *Rlim* gene, thereby knocking out the gene.

**Figure 3:**
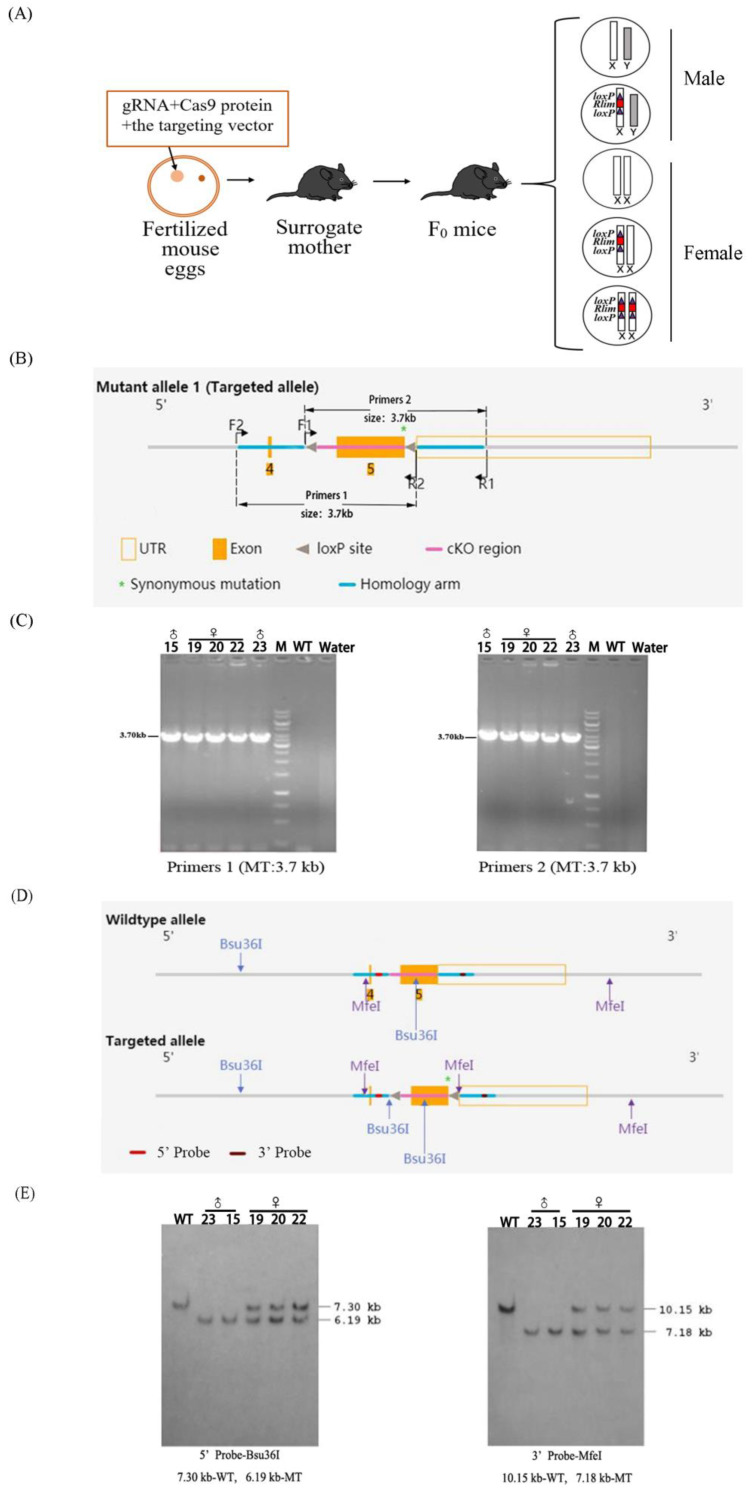
Production and identification of F_0_ generation *Rlim*^fl^ transgenic male mice and *Rlim*^fl/+^ transgenic female mice. (**A**) Schematic diagram of the production of five different types of possible F_0_-generation mice by microinjection of fertilized eggs. (**B**) Schematic diagram of primer positions for PCR identification of transgenic mice. The PCR product length of both Primer 1 and Primer 2 are 3.7 kb. (**C**) PCR identification results of F_0_-generation transgenic mice. #15, #19, #20, #22 and #23 mice were identified as F_0_ generation transgenic mice. (**D**) Schematic diagram of the recognition sites of restriction enzymes and the location of DNA probes used in the Southern blotting analysis. To employ the Bsu36I site for Southern blotting analysis, an exogenous Bsu36I site was introduced between the left homologous arm and the left *loxP* site in the targeted allele. (**E**) Southern blot identification results in F_0_ generation transgenic mice. #15, #19, #20, #22 and #23 mice were identified as F_0_ generation transgenic mice. WT represents wild type and MT represents the mutant allele.

We microinjected two *Rlim* targeting sgRNAs, along with the Cas9 protein and the targeting vector, into fertilized mouse eggs and transferred the injected embryos into the oviducts of surrogate mother mice. Theoretically, F_0_ mice with five different possible genotypes could be produced from the surrogate mother mice (Figure 3A). After genotyping the F_0_-generation mice by PCR, two male *Rlim*^fl^ transgenic mice and three female *Rlim*^fl/+^ transgenic mice were identified (Figure 3B,C). These five F_0_-generation transgenic mice were further confirmed by Southern blotting analysis (Figure 3D,E).

### 3.2. Production and Identification of Homozygous Rlim^fl/fl^ and Heterozygous Rlim^fl/+^ Transgenic Female Mice

**Figure 4:**
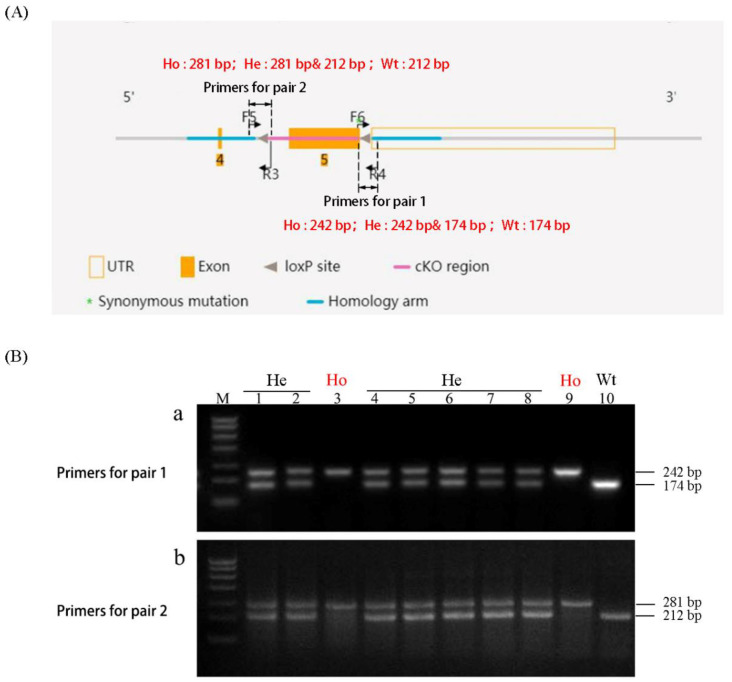
Identification of homozygous *Rlim*^fl/fl^ and heterozygous *Rlim*^fl/+^ transgenic female mice. (**A**) Schematic diagram of primer locations for PCR identification of homozygous *Rlim*^fl/fl^ and heterozygous *Rlim*^fl/+^ transgenic female mice. The PCR products of primer pair 1 (F6 and R4) for homozygous transgenic, heterozygous transgenic, wild-type mice are 242 bp (contains the right *loxP* site), 242 bp and 174 pb, 174 bp (no *loxP* site) in length, respectively. The PCR products of primer pair 2 (F5 and R3) for homozygous transgenic, heterozygous transgenic, and wild-type mice are 282 bp (contains the left *loxP* site), 282 bp and 212 bp, 212 bp (no *loxP* site) in length, respectively. (**B**) PCR identification results of transgenic female mice. Ho represents homozygotes; He represents heterozygotes; Wt. represents wild type.

The male *Rlim*^fl^ transgenic mice were mated with female *Rlim*^fl/+^ transgenic mice, and their female offspring were genotyped by PCR. An expected ratio of homozygous *Rlim*^fl/fl^ and heterozygous *Rlim*^fl/+^ transgenic female mice were identified (Figure 4A,B). The homozygous *Rlim*^fl/fl^ transgenic female mice were used for subsequent *Rlim* knockout experiments, while their heterozygous sisters were used as controls.

### 3.3. Knockout of the Maternal Rlim Allele in Embryos Resulted in Offspring Sex-Ratio Shift toward Males but Superovulation in the Mothers Did Not Compensate for the Reduced Litter Size

**Figure 5:**
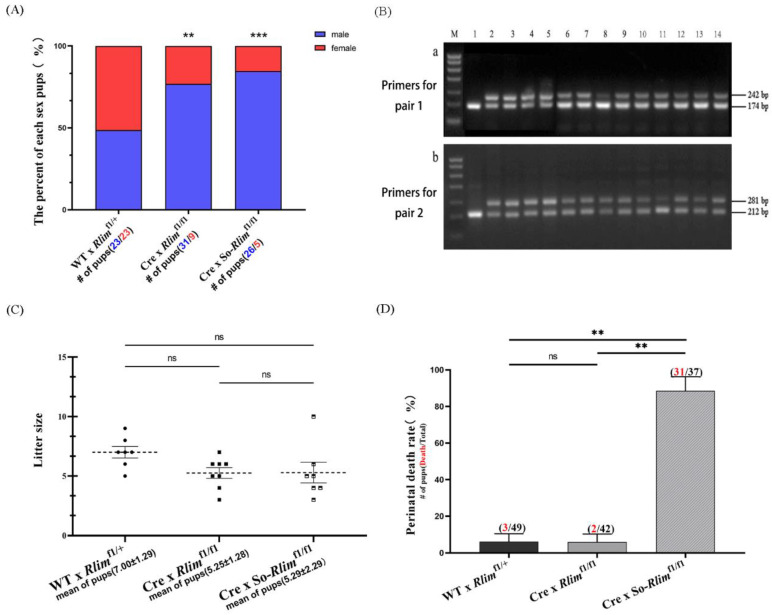
Effects of superovulation in the mother mice and maternal *Rlim* allele knockout in the embryos on the sex ratio of offspring, litter size and perinatal motility of the offspring. (**A**) Effects of knocking out the embryo’s maternal *Rlim* allele on the sex ratio of born offspring. When the transgenic father mice carry an X-linked Cre-enzyme gene and the homozygous *Rlim*^fl/fl^ transgenic mother mice mate, the maternal *Rlim* allele is knocked out in the female embryos. (**B**) Genotype identification of the 14 female-born offspring mice delivered by the homozygous *Rlim*^fl/fl^ transgenic mother mice mated with the Cre-transgenic father mice. When amplifying the genomic DNA of the 14 female-born offspring mice using primers for pair 1 and primers for pair 2, since both primers F6 and R3 were located on the *Rlim* gene knockout region (see Figure 4A), the heterozygous *Rlim*^KO/+^ knockout female offspring mice (with a successful knockout of the maternal *Rlim* allele) will not show the 242 bp band and the 282 band, respectively. It will, however, show the 174 bp band and the 212 bp band, respectively. Heterozygous *Rlim*^fl/+^ transgenic female offspring mice (without successful knockout of the maternal *Rlim* allele) will show 242 bp and 174 pb bands, and 281 bp and 212 pb bands, respectively. (**C**) Effects of superovulation in the mother mice and knockout of the maternal *Rlim* allele in the embryos on litter size. (**D**) Effects of superovulation in the mother mice and knockout of the maternal *Rlim* allele in the embryos on the perinatal death rate of born progenies. The number of perinatal deaths was calculated by the number of stillborn pups and the number of pups that died within 3 days after birth. So represents superovulated *Rlim*^fl/fl^ mother mice, ns represent nonsignificant difference at *p* > 0.05; ** and *** represent *p* < 0.01 and *p* < 0.001, respectively.

To investigate the effects of inactivating the maternal *Rlim* allele in female embryos on the sex ratio of offspring, we crossed superovulated and non-superovulated *Rlim*^fl/fl^ females with transgenic males carrying a CMV-Cre enzyme gene on the X chromosome. Their offspring had a significantly shifted sex ratio toward males (Figure 5A). This suggests that knocking out the maternal *Rlim* allele impairs the development of female embryos, resulting in a male-biased sex ratio skew in the progeny. Despite the reduced ratio of female pups born following mutation of the maternal *Rlim* allele in female embryos, we still observed 14 female pups (Figure 5A). According to the genotyping of these 14 female pups, one carried a mutated maternal *Rlim* allele, while the remaining 13 carried a maternal *Rlim* allele with two integrated *loxP* sites (Figure 5B), indicating that their maternal *Rlim* allele was not deleted.

Although knocking out the maternal *Rlim* allele in female embryos shifted the offspring sex ratio toward males, it decreased the litter size (Figure 5C). Superovulation in the *Rlim*^fl/fl^ mothers mated with CRE-enzyme transgenic male mice failed to compensate for the reduced litter size (Figure 5C). More surprisingly, superovulation treatment on the *Rlim*^fl/fl^ females crossed with Cre-transgenic males significantly increased the perinatal mortality of the born offspring (Figure 5D).

### 3.4. Superovulation in the Mother Mice Increased the Perinatal Death Rate of the Progeny

**Figure 6:**
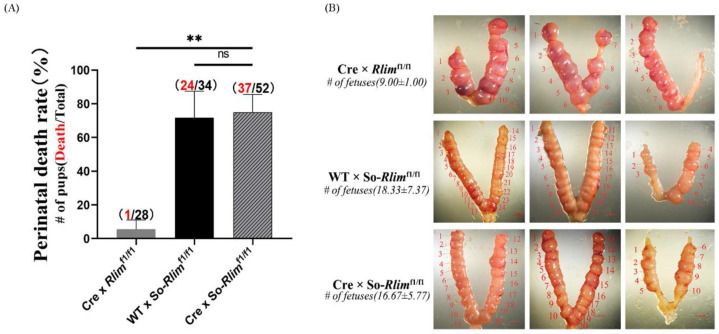
Superovulation in the mother mice increased the perinatal mortality of pups. (**A**) Effects of superovulation in the mother mice on the perinatal death rate of the offspring. Red marks the total number of pups that died during the perinatal period and black marks the total number of pups that were born. (**B**) Effects of superovulation in the mother mice on the implantation of E7.5–E8.5 fetuses. ns represents nonsignificant difference at *p* > 0.05; ** represents *p* < 0.01.

Whether the high perinatal death rate seen in the progenies of superovulated *Rlim*^fl/fl^ females mated with CRE-enzyme transgenic males was caused by superovulation in the mothers, or the combined effects of superovulation in the mothers and inactivating the maternal *Rlim* allele in the female embryos is unknown. To find out the answer, we performed superovulation experiments on *Rlim*^fl/fl^ female mice that were crossed with CRE-enzyme transgenic male mice or with wild-type male mice. The results showed that in both groups of superovulated mother mice, the offspring’s perinatal mortality was significantly increased (Figure 6A). This suggests that superovulation treatment on the mother mice impairs the perinatal development of offspring independent of maternal *Rlim* allele deletion.

To determine whether superovulation affects the development of the fetuses, we harvested the uteruses of superovulated pregnant mother mice on embryonic days E7.5–E8.5 and analyzed the number and development of the fetuses. Although superovulation increased the number of embryos, it also impaired embryo growth, as the size of embryos in two groups of superovulated mothers was significantly smaller than that in the control group (Figure 6B). This result suggests that superovulation impairs embryonic development in utero, thus increasing perinatal mortality in the offspring.

### 3.5. RNA Interference of Rlim in Embryos Resulted in Production of Male-Only Offspring

We also tested the effects of RNA interference of *Rlim* in mouse embryos on offspring sex ratio. Transfer of fertilized eggs that were microinjected with *Rlim* siRNA into recipient mothers resulted in the birth of male-only progenies, which all normally survived into adulthood (Table 3). This suggests that siRNA-mediated knockdown of *Rlim* also inhibits the onset of imprinted XCI, thereby causing the death of female but not male embryos in utero.

## 4. Discussion

**Figure 7:**
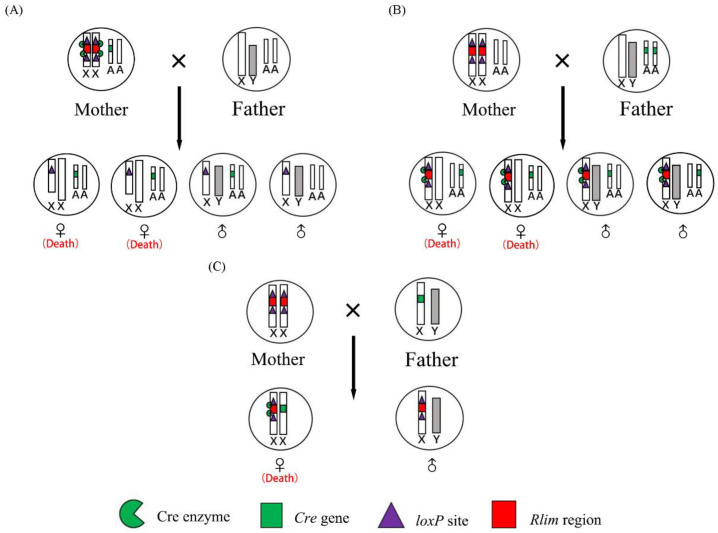
Schematic diagram of three different strategies using the CRE-*loxP* system to knockout the maternal *Rlim* gene in female embryos. (**A**) Knockout of the maternal *Rlim* gene in female embryos by mating wildtype fathers with *Rlim*^fl/fl^ mothers carrying an autosome-linked *Cre* transgene driven by an oocyte-specific promoter. (**B**) Knockout of the maternal *Rlim* gene in female embryos by mating *Rlim*^fl/fl^ mothers with fathers carrying a Rosa 26 promoter-controlled *Cre* transgene on autosomes. (**C**) Knockout of the maternal *Rlim* gene in female embryos by mating *Rlim*^fl/fl^ mothers with transgenic fathers carrying a *CMV-Cre* gene integrated on the X chromosome. Knockout of the maternal *Rlim* gene in male embryos will not affect their growth.

Different strategies can be used to inactivate the maternal *Rlim* allele in female embryos. In two previously reported papers, the authors used a *Cre* transgene inserted on a maternal autosome [18] or a paternal autosome [18] to delete the maternal *Rlim* gene in female embryos (Figure 7A,B). These two approaches produced male offspring carrying an autosome-linked *Cre* transgene and a null maternal *Rlim* gene. However, the *Cre* transgene causes toxic effects on the male host animals by inducing genomic DNA recombination between two pseudo *loxP* sites, which have been found in the mammalian genome [23,24,25,26]. Moreover, the mutated maternal *Rlim* gene reduces the number and motility of sperm in adult male mice [20]. Hence, inactivating the *loxP*-flanked maternal *Rlim* gene with an autosome-linked *Cre* transgene in female embryos will cause toxicity and subfertility problems to their brothers. In this study, we used a *Cre* transgene integrated on the paternal X chromosome to delete the maternal *Rlim* allele in female embryos. The male progeny resulting from this strategy only carry a *loxP*-flanked *Rlim* gene, which have no obvious negative effects on the host male animals. Therefore, the strategy used in this study for inactivating the female embryo’s maternal *Rlim* allele is better than those mentioned above.

Female mouse embryos with a deleted maternal *Rlim* allele all die in utero and are resorbed before birth due to defective placental development caused by abnormal imprinted XCI [18,22]. However, we found in this study that a liveborn female mouse carries a mutated maternal *Rlim* allele. This female most likely was a chimera that carried a null maternal *Rlim* allele in the PCR-tested tail tissue, but with an intact *loxP*-flanked maternal *Rlim* allele in the untested placental tissue, which allowed the chimera female embryo to normally develop to term. We also found that *Rlim*^fl/fl^ females crossed with *Cre* transgenic males produced 13 live female offspring with intact *loxP*-flanked maternal *Rlim* allele. These 13 females should also carry a paternal X chromosome-linked *CMV-Cre* transgene inherited from their fathers. Why in these 13 female-born offspring did the *CMV-Cre* transgene not induce deletion of the *loxP*-flanked maternal *Rlim* gene? This might be explained by the fact that in these 13 females, before a sufficient amount of CRE enzyme could be synthesized to delete the *loxP*-flanked maternal *Rlim* gene, the paternal X chromosome-linked *CMV-Cre* transgene was silenced by an imprinted XCI, which occurs on the paternal X chromosome of female embryos at about the four-cell stage [18,22]. Nevertheless, in those non-viable female embryos carrying a null maternal *Rlim* gene, enough amount of CRE enzyme was expressed from the *CMV-Cre* transgene and the maternal *Rlim* gene was successfully inactivated before imprinted XCI occurs.

We demonstrated that it is not only the *CMV-Cre* transgene-mediated knockout of *Rlim*, but also the siRNA-based knockdown of *Rlim* in embryos that causes a male-biased sex ratio shift in the offspring. However, the former approach resulted in a lower male-to-female ratio in the offspring than the later method. This is probably due to the fact that the *CMV-Cre* transgene starts to transcribe at the two-cell stage following embryonic genome activation, and thus inactivates the *Rlim* gene at, or more possibly after, the two-cell stage, but the microinjected siRNA suppresses *Rlim* expression at the one-cell stage. Hence, in female embryos, the imprinted XCI initiating at about the four-cell stage is not blocked completely by the *CMV-Cre* transgene-mediated knockout of *Rlim*, but fully inhibited by the siRNA-based knockdown of *Rlim*. The former method results in the birth of a small number of female progenies, but the later approach leads to no birth of female offspring, as observed in the present study.

It has been reported that superovulation in female mice leads to the expulsion of some low-quality oocytes [27,28] and produces an excessive number of embryos, which causes overload of the uterus [29,30] and impairment of fetal growth in utero [21,31]. The prenatal detrimental effects caused by superovulation in the mothers might increase the perinatal mortality of newborn mice. Similar results were observed in this study. Female mouse embryos with a mutated maternal *Rlim* allele die and are resorbed during embryonic days E7.5 to E11.5 [18]. Superovulated *Rlim*^fl/fl^ dams mated with *Cre* transgenic males may have also carried an excessive number of embryos by embryonic days E11.5, thus resulting in uterine overload, poor fetal development and ultimately a high perinatal death rate in newborn mice.

Gender ratio can be controlled by preselecting the sperm or the embryos. Using the former method for sex ratio control does not negatively influence the litter size [17,32,33]. Nevertheless, the later approach is known to decrease the litter size [15,16], which may not be compensated by superovulation, as observed in this study. Therefore, sex control technologies based on preselecting sperm might be more promising than those based on preselecting embryos.

## 5. Conclusions

In summary, knocking out the *loxP*-flanked maternal *Rlim* allele in female mouse embryos via a paternal X-linked CMV-Cre transgene resulted in a male-biased sex ratio shift in the offspring, but the reduced litter size caused by the loss of female embryos could not be compensated by superovulation in the mother mice. This study provides a new strategy for male-biased gender selection.

## Figures and Tables

**Table 1:** Sequences of gRNAs, *Rlim*-siRNA and NC-siRNA used in this study.

Name	Sequence (5′~3′)
gRNA1	AAACTACATCATCATAGTCGGGG
gRNA2	GCAGGGCAGTCTTATCTTCTGGG
*Rlim*-siRNA	GAAGUCAAAUGGAUCGCUUTT
	AAGCGAUCCAUUUGACUUCTG
NC-siRNA	UUCUCCGAACGUGUCACGUTT
	ACGUGACACGUUCGGAGAATT

**Table 2:** Sequences of primes and probes used in this study.

Name	Forward Primer	Reverse Primer	Amplicon Size
Primers 1	ACGTAAACGGCCACAAGTTC	AGAGTACTGGGGTTATCACAATCT	3.7 kb
Primers 2	CTATGCATCTGGGTACAAAATAACC	GTGGATTCGGACCAGTCTGA	3.7 kb
5′Probe-Bsu36I	ACTGCTGTGTCTGCCTCACCTTTG	AGAGAAGCACCATTCCCCAGCATA	WT-7.30 kb MT-6.19 kb
3′Probe-MfeI	AAAGGAAAGGACCGTGCAGAACC	CCCAAGAAAGCTCTGCCAAATGTACT	WT-10.15 kb MT-7.18 kb
Primers for pair 1	TTGTCGCAGGGCAGTCTTATC	GCAATGACTCAATTCAGCTTGTGA	Homozygotes: 242 bp Heterozygotes: 242 bp and 174 bp Wildtype allele: 174 bp
Primers for pair 2	AGCCTTGTTTATAGTTTTGCTCTGG	GCTGTGGGAAGGCATGAATTTT	Homozygotes: 281 bp Heterozygotes: 281 bp and 212 bp Wildtype allele: 212 bp
*CMV-Cre*	GTAGGCGTGTACGGTGGGAGGT	TCCAGGTATGCTCAGAAAACGCC	349 bp
SRY	CTTTTTCCAGGAGGCACAGA	GACAGGCTGCCAATAAAAGC	250 bp
ZFX	AAGAGAGTCCATTCAAGTGTGA	GCTACCTTTGTTGCCGAAAT	399 bp

**Table 3:** Effects of RNA interference of *Rlim* in mouse embryos on offspring sex ratio.

Groups	No. of Transferred Injected Embryos/Recipient Mothers	No. of Born Pups/Survive into Adulthood	Male:Female
NC-siRNA	22/2	10/10	4:6
*Rlim*-siRNA	22/2	12/12	12:0 ***

NC represent negative control; *** represent *p* < 0.01.

## Data Availability

The data that support this study are available within the article and available from the authors upon request.
